# Galvanic vs. pulsatile effects on decision-making networks: reshaping the neural activation landscape

**DOI:** 10.1088/1741-2552/ad36e2

**Published:** 2024-04-03

**Authors:** Paul W Adkisson, Cynthia R Steinhardt, Gene Y Fridman

**Affiliations:** 1 Department of Biomedical Engineering, Johns Hopkins University, Baltimore, MD 21205, United States of America; 2 Department of Otolaryngology Head and Neck Surgery, Johns Hopkins University, Baltimore, MD 21205, United States of America; 3 Center for Theoretical Neuroscience, Columbia University, New York, NY 10027, United States of America; 4 Simons Society of Fellows, Simons Foundation, New York, NY 10010, United States of America

**Keywords:** galvanic, pulsatile, neuromodulation, tDCS

## Abstract

**Objective.:**

Primarily due to safety concerns, biphasic pulsatile stimulation (PS) is the present standard for electrical excitation of neural tissue with a diverse set of applications. While pulses have been shown to be effective to achieve functional outcomes, they have well-known deficits. Due to recent technical advances, galvanic stimulation (GS), delivery of current for extended periods of time (>1 s), has re-emerged as an alternative to PS.

**Approach.:**

In this paper, we use a winner-take-all decision-making cortical network model to investigate differences between pulsatile and GS in the context of a perceptual decision-making task.

**Main results.:**

Based on previous work, we hypothesized that GS would produce more spatiotemporally distributed, network-sensitive neural responses, while PS would produce highly synchronized activation of a limited group of neurons. Our results in-silico support these hypotheses for low-amplitude GS but deviate when galvanic amplitudes are large enough to directly activate or block nearby neurons.

**Significance.:**

We conclude that with careful parametrization, GS could overcome some limitations of PS to deliver more naturalistic firing patterns in the group of targeted neurons.

## 1. Introduction

An accepted theoretical model of how we reach a decision given two alternatives posits that the average firing rates of two opposing neural populations encode the saliency of each alternative, and a decision is made when one of the two populations clears some firing rate threshold [1–3]. Using a well-accepted *in-silico* model of this process, we asked how electrical stimulation of one of the neural populations would influence the decision-making process. This problem is nontrivial because of the recurrent nature of the network with excitatory and inhibitory interconnections. We compared two forms of electrical stimulation each applied to a well-known decision-making task: conventional pulsatile stimulation (PS) and more recently re-introduced, galvanic stimulation (GS).

Biphasic PS is the standard for safe and effective electrical stimulation of the brain. In basic research, low-amplitude (e.g. 5 *μ*A) sub-millisecond pulses or sequences of pulses are commonly used to probe brain connectivity and function [4–7]. Clinically, pulses are used in neural prosthetics [8], preresection surgeries for drug-resistant epilepsy [9], deep brain stimulation for Parkinson’s disease [10], and a host of emerging applications in ‘bioelectronic medicine’ [11]. While pulses are clearly capable of inducing sensory percepts [8], muscle movements [12], and bias decision making [13], there are clear limitations to their ability to deliver natural sensation [14] or motion [15].

GS (a.k.a. ‘direct current’ or DC) has been limited to transcutaneous application (such as transcranial direct current stimulation or tDCS), due to safety implications associated with electrolysis and pH changes at the metal electrode [16, 17]. This limitation however is being addressed in the recent development of implantable GS devices [17, 18] and conventional stimulators with high capacity electrodes [19].

Pulses delivered to an electrode implanted near isolated neurons will cause most of the neurons within the targeted region to evoke an action potential (AP) in phase with the presented pulse. This rule-of-thumb response however does not hold for neurons located very close to the electrode or those located further away. Nearby neurons may be blocked from evoking an AP and those located further away will experience only slight depolarization that does not result in an AP. The interaction with the naturally occurring ‘spontaneous’ activity is more complex since the neuron can be in the refractory period when the pulse arrives and result in that pulse being ineffective in being able to evoke an AP [20]

In contrast, by smoothly altering the extracellular potential, GS changes a neuron’s membrane potential, with cathodic GS (CGS) bringing it closer or anodic GS (AGS) further from the AP generation threshold. As a result, GS modulates firing rates up and down while preserving natural firing statistics and without producing unnatural synchrony due to phase-locking [21–24].

The single neuron effects of PS and GS lead to hypotheses of how a network of excitatory and inhibitory neurons would behave. We hypothesize that a population of neurons experiencing GS will be more sensitive to network level excitation and inhibition than if this population were subjected to PS. The ability of a network to reach persistent activity states is critical in maintaining working memory for perceptual decision making [2]. We also hypothesize that GS activation will be spread more uniformly throughout the stimulated population, while PS may have high population-wide variability between high firing rate neurons and low firing rate neurons. We further hypothesize that PS will induce unnaturally high levels of neural synchrony which may lead to different decision-making latencies.

We extended a well-established computational model of perceptual decision making [2] to explore these predictions by adding pulsatile and GS to bias the decision-making output of the model. The investigation in this manuscript is a rigorous exploration of the cortical network effects previously suggested in our EMBC conference proceedings publication [25]. To add considerably more explanation to the phenomenological observations described in that work, here we explore the comparison based on a ‘level playing field’ set up by behavioral equivalence between PS and CGS, and examine the inhibitory effects of AGS.

## 2. Materials and methods

### 2.1. Realistic intracortical microstimulation model

For the sake of computational efficiency, the perceptual decision-making model extended here [2] approximates individual neuron behavior with leaky-integrate-and-fire (LIF) dynamics. Each LIF neuron is modeled by

$$\frac{\text{d}V_{m}}{\text{d}t}=\frac{-g_{L}\left(V_{m}-E_{L}\right)+I_{\text{syn}}+I_{\text{stim}}}{C_{m}};V_{m}<V_{\text{threshold}}.$$

Once $V_{m}$ exceeds $V_{\text{threshold}}$, an AP is recorded, $V_{m}$ is reset to $V_{r}$, and that neuron goes into a refractory period for $\tau_{r}$ ms. axon (see supplementary material table S1 for the parameter values).

Although this simplification yields accurate population-averaged neural firing rates under physiological conditions, it does not naturally accommodate intracortical electrical microstimulation.

The challenge that arises when representing extracellular current stimulation with LIF neurons is that LIF neurons only contain a single membrane potential representing the whole ‘point’ neuron whereas biological neurons are spatially extended with a gradient of different membrane potentials depending on the location relative to the electrode. To address this challenge, we assume that the magnitude of the voltage response to extracellular current stimulation is greatest in the axon segment that is closest to the electrode. We then defined the point-location of each LIF neuron as being localized at this axon segment.

We estimated the current $I_{\text{stim}}$ injected into the LIF neuron based on the distance from the neuron to the electrode. The electrical current at the electrode $I_{\text{electrode}}$ creates an electric field around the electrode, such that extracellular voltage at any point distance r from the electrode is given by

$$V_{\text{ext}}(r)=\frac{\rho_{\text{ext}}}{4\pi r}I_{\text{electrode}}$$

where $\rho_{\text{ext}}$ is the uniform extracellular tissue resistivity.

Then, based on the mirror estimate [26], the steady-state membrane potential can be expressed as a function of the extracellular voltage

$$V_{m}^{\text{ss}}=\bar{V_{\text{ext}}}-V_{\text{ext}}$$

where $\bar{V_{\text{ext}}}$ is the average extracellular voltage across all the spatial nodes along a linearly positioned axon (see supplementary material for details).

$I_{\text{stim}}$ for a given LIF neuron is then assumed to be proportional to $V_{m}^{\text{ss}}$ at the location of the LIF point neuron, and therefore corresponding to the maximum membrane potential for the biological neuron at that location:

$$I_{\text{stim}}=k_{\text{gs}}V_{m}^{\text{ss}}\text{and}I_{\text{stim}}=k_{\text{ps}}V_{m}^{\text{ss}}$$

for galvanic and PS, respectively.

In the case of GS, the current is delivered for the duration of the stimulation. In case of PS, the current is delivered for the duration of the pulse presentation.

We estimated the values for $k_{\text{gs}}$ and $k_{\text{ps}}$ by matching the LIF responses to a cable equation simulation of a linear axon positioned near an electrode source [17] axon (see supplementary material for Cable Equation and table S4 for parameter values). We then uniformly distributed the neurons around the electrode from 10 *μ*m–2 mm away based on neural mapping of staining work by Levitt *et al* [27].

### 2.2. Pulsatile and galvanic blocking effects

In further examination of the previously observed neural responses to PS [28], our recent work [29, 30], using an adapted axon model from Hight and Kalluri [31], systematically catalogues the possible firing rate responses to biphasic PS trains. For example, pulses can block the spontaneous APs, spontaneous APs can block subsequent pulses, and pulses can block subsequent pulses. These refractory effects are amplitude-dependent, with higher-amplitude pulses causing longer blocking periods. They are also dependent on the spontaneous firing rate, with higher spontaneous firing rates resulting in more pulse-spontaneous interactions. We therefore expect that delivering PS to a population of neurons would induce a mosaic of activation and deactivation, since each neuron in a network experiences a different spontaneous firing rate and a different maximum extracellular voltage based on its distance to the electrode. We approximated these effects by adding pulse-pulse $\left(t_{\text{pp}}\right)$ and pulse-spontaneous $\left(t_{\text{ps}}\right)$ refractory periods analogous to the spontaneous refractory period inherent in the LIF model.

During these refractory periods, membrane potential is freely allowed to vary, but pulse induced APs are not initiated during the pulse-pulse refractory period, and spontaneous APs are not initiated during the pulse-spontaneous refractory period. To capture the amplitude-dependence of pulse-pulse blocking effects, we used the blocking times $\left(t_{\text{pp}}\right)$ from Steinhardt and Fridman [29] (0–132 ms) parameterized at various extracellular amplitudes; we generated intermediate values using linear interpolation to produce a continuous refractory function. If a pulse arrived while a neuron was already in the pulse-pulse refractory period, a refractory time of $\frac{t_{\text{pp}}}{2}$ was used to capture residual pulse-pulse blocking effects. (see supplementary materials table S6 for details)

Using this approach, we replicated the characteristic amplitude dependence and spontaneous rate dependence in the pulse rate vs. firing rate curves [29].

Steinhardt and Fridman [24] also quantified the depolarizing block that can occur with excessive CGS. This effect is also dependent on the spontaneous firing rate, with higher spontaneous rates inducing block at lower cathodic amplitudes. We adapted this effect to the LIF model by setting an instantaneous maximum current during GS (1135 pA) such that steady state membrane potential could not exceed −19 mV. This was based on the observation that membrane potential stabilizes at −19 mV at the threshold current of depolarizing block from Qian *et al* [32]. Anytime instantaneous current inputs exceeded this maximum, the input currents were manually set to 0 pA. With this simple rule, we replicated the characteristic amplitude and spontaneous rate dependence of GS. Importantly, our amplitudes are consistent with those observed in nerve blocking studies [33].

### 2.3. Biophysical attractor model

**Figure 1.**
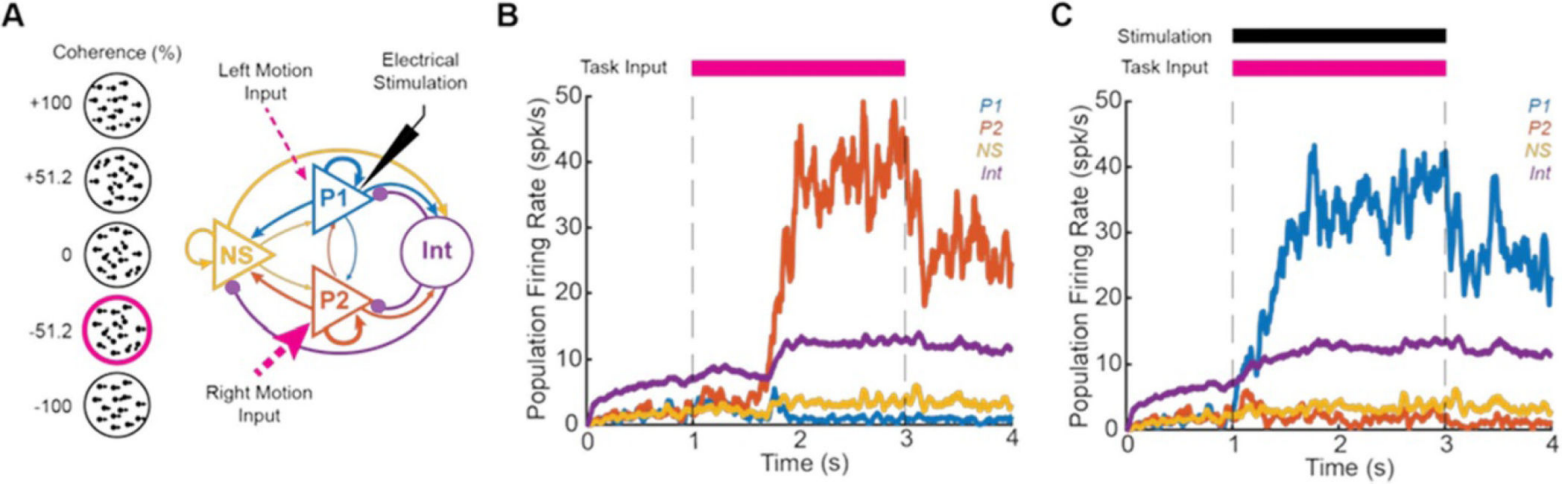
Experimental Design. (A) Model consists of two subpopulations (P1 and P2) responsive to leftward and rightward motion, non-selective pyramidal neurons (NS) and inhibitory interneurons (Int). Neurons are connected with strong, medium, and weak connections (thickness proportional to strength), with purple arrows indicating inhibitory connections. During a trial, all neurons receive background input. From 1–3 s, P1 and P2 receive task-related input proportional to coherence of left versus rightward motion. P1 also receives electrical stimulation from 1–3 s (black) to bias the network behavior. (B) Mean population firing rates of P1 (blue), P2 (red), NS (yellow), and Int (purple) in a representative control trial without electrical stimulation. Task input strongly favors P2 (−51.2% coherence) resulting in P2 winning the trial. (C) Mean population firing rates in a representative trial with PS of P1. Despite task input favoring P2 (−51.2% coherence), PS biases the network such that P1 wins the trial.

The biophysical model was based on a well-established decision-making network [2]. The model simulates a two alternative forced choice task with P1 (blue) and P2 (red) encoding task input (strength of moving dot leftward versus rightward motion). A non-selective (NS) population (yellow) and inhibitory interneuron (Int) population (purple) are also included for a winner-take-all network construction (figure 1(A)). The network model consisted of N neurons (80% pyramidal neurons and 20% inhibitory interneurons), connected with weights:

$$w_{\text{weak}}=0.8765,w_{\text{medium}}=1,w_{\text{strong}}=1.7$$

for weak, medium, and strong connections respectively (figure 1, strength shown with line thickness).

Importantly, all neurons in this model were connected with one of these three weights. The model simulated neurons with LIF dynamics and synaptic currents from AMPA, NMDA, and GABA receptors. As described in [2], synaptic currents are modeled by

$$I_{\text{syn}}=\sum_{n=1}^{N_{E}}w_{n}I_{\text{AMPA}}^{\left(n\right)}+\sum_{n=1}^{N_{\text{Int}}}w_{n}I_{\text{GABA}}^{\left(n\right)}+\sum_{n=1}^{N_{E}}w_{n}I_{\text{NMDA}}^{\left(n\right)}$$

where $w_{n}$ is the weight of the connection (strong, medium, or weak). For the following equations we consider a single synapse of each type.

AMPA currents are exponential decay functions with external and recurrent components

$$I_{\text{AMPA}}=\left(g_{{\text{AMPA}}_{\text{ext}}}s_{{\text{AMPA}}_{\text{ext}}}+g_{{\text{AMPA}}_{\text{rec}}}s_{{\text{AMPA}}_{\text{rec}}}\right)\left(E_{\text{AMPA}}-V_{m}\right)\;\frac{\text{d}s_{\text{AMPA}}}{\text{d}t}=-\frac{s_{\text{AMPA}}}{\tau_{\text{AMPA}}}$$

where $s_{{\text{AMPA}}_{\text{ext}}}$ is incremented every time an external AP is recorded, and $s_{{\text{AMPA}}_{\text{rec}}}$ is incremented every time a recurrent AP is recorded with synaptic delay $\tau_{\text{delay}}$. Note, external or background input currents represent inputs to the network from outside neurons such as upstream visual neurons. Recurrent APs refer to synaptic inputs from within the network that is being modeled.

GABA currents are exponential decay functions with different constants

$$I_{\text{GABA}}=\left(g_{\text{GABA}}s_{\text{GABA}}\right)\left(E_{\text{GABA}}-V_{m}\right)\;\frac{\text{d}s_{\text{GABA}}}{\text{d}t}=-\frac{s_{\text{GABA}}}{\tau_{\text{GABA}}}$$

where $s_{\text{GABA}}$ is incremented every time a recurrent inhibitory AP is recorded with synaptic delay $\tau_{\text{delay}}$.

NMDA currents are double exponential functions with both a rise time constant and a decay time constant, as well as a magnesium-dependent conductivity constant.

$$I_{\text{NMDA}}=\frac{\left(g_{\text{NMDA}}s_{\text{NMDA}}\right)\left(E_{\text{NMDA}}-V_{m}\right)}{1+\frac{C_{\text{Mg}}e^{-62V_{m}}}{3.57}}\;\frac{\text{d}x_{\text{NMDA}}}{\text{d}t}=-\frac{x_{\text{NMDA}}}{\tau_{{\text{NMDA}}_{1}}}\;\frac{\text{d}s_{\text{NMDA}}}{\text{d}t}=\alpha x_{\text{NMDA}}\left(1-s_{\text{NMDA}}\right)-\frac{s_{\text{NMDA}}}{\tau_{{\text{NMDA}}_{2}}}$$

where $x_{\text{NMDA}}$ is incremented every time a recurrent excitatory AP is recorded with synaptic delay $\tau_{\text{delay}}$. Note, $V_{m}$ is expressed in volts rather than mV.

For our simulations, N=1000 neurons and a time step of dt=0.05 ms were used. (see supplementary material tables S2 and S3 for the parameter values).

### 2.4. Perceptual decision-making task

A random dot motion task was simulated at coherence levels from fully leftward (+100%) to fully rightward (−100%) coherence (figure 1(A), circles). 100 trials were executed at each coherence level under four conditions: PS, CGS, AGS, and no-stimulation control. Throughout each 4-second trial, all neurons received 2400 Hz background Poisson inputs that triggered AMPA EPSCs. This caused the pyramidal neurons in the network to fire spontaneously at 2–3 spks/s (figure 1(B)). At t=1s, neurons in populations P1 (blue) and P2 (red) received task-related input proportional to c:

$${\text{FR}}_{\text{task}}=40c+40$$

where if motion is in the opposite direction c is negative [2] (figures 1(A) and (B), magenta). To bias the network, all neurons in P1 also received electrical stimulation (black) concurrent with task input (figure 1). At t=3s, task-related inputs and stimulation ceased. Decision making experiments show a sigmoidal relationship between coherence and accuracy. To capture this relationship, coherences were sampled logarithmically around the empirically determined center of the sigmoid for each stimulation condition. For control stimulation, this was 0% [2, 34]. For pulsatile and CGS, the center coherence was estimated as −57.0%. For AGS, the center coherence was estimated as +30.0%.

### 2.5. Decision data analyses

Instantaneous neuron firing rates were calculated in 5 ms bins, followed by a 50 ms moving average. Population firing rates were then taken as the average instantaneous firing rate of all the neurons in each subpopulation. Decisions were recorded at the end of the 4-second trial, if the final average firing rate of one of the two neural subpopulations (P1 or P2) exceeded 15 spk/s, while the other did not. In such cases, the subpopulation whose firing rate exceeded 15 spk/s was deemed the ‘winner’ of the trial. This threshold was chosen based on Wang [2]. Decision data were then analyzed using logistic regression as in Hanks *et al* [34]. Differences in decision making were assessed for significance by 2-sample non-parametric bootstrap (N=10000) on the bias and sensitivity parameters. The time at which the winning subpopulation exceeded 15 spk/s after the start of task stimulation (t=1s) was considered the decision time. Trials that reached their decision after the task period (t>3s) were excluded from analyses of time-dependent effects (figures 3–5). Differences in decision times were assessed for significance by unpaired t-tests.

### 2.6. Disconnected, feedback only, and recurrent only network analyses

Task firing rates were computed by binning all spikes for each P1 neuron between t=1 and t=3s. In the disconnected condition, P1 neurons were not connected to any other neurons in the network. In the feedback only condition, P1 neurons were connected with 30 inhibitory interneurons (keeping an 80/20 E/I ratio) with standard synaptic strength w=1. In the recurrent excitation only condition, P1 neurons were connected to one another with weakened synaptic strength (w=1). Population-averaged task firing rates were computed by averaging all the individual neuron task firing rates in P1. All stimulation conditions were compared relative to control by subtracting the mean of the control group. Differences in distributions of firing rates across stimulation conditions were assessed for significance by Kolmogorov–Smirnov tests. Differences in individual neural firing rates were assessed for significance by 1-tailed t-tests with Bonferroni correction for multiple comparisons. Differences in population-averaged task firing rates were assessed for significance by Kruskal–Wallis tests.

### 2.7. Firing rate trajectory data analyses

Start-of-task firing rates were computed by binning all spikes from P1 neurons occurring between t=1 and t=1.1s and dividing by the duration (0.1 s). End-of-task firing rates were computed similarly for the period between t=2.9 and t=3.0s. The firing rate slope around the decision-making threshold (15 spk/s) was estimated separately for each trial as

$$m=\frac{20-10}{t_{20}-t_{10}},$$

where $t_{10}$ is the first time when the P1 population firing rate exceeded 10 spk/s and $t_{20}$ is the first time the P1 population firing rate exceeded 20 spk/s. This method mitigated noise in the P1 population firing rates. The maximum P1 firing rates were computed by finding the maximum instantaneous firing rate in the P1 population for each trial. All stimulation conditions were compared relative to control by subtracting the mean of the control group. All comparisons of P1 firing rate trajectories were assessed for significance by Kruskal–Wallis tests.

### 2.8. Firing rate distribution data analyses

Instantaneous neuron firing rates were calculated in 50 ms bins, followed by a 200 ms moving average. Firing rates were down sampled to 20 Hz to match temporal resolution. Kurtosis of the resulting firing rate trace was calculated for each time point and trial separately. Beginning (0–0.5 s) and end (3.5–4 s) of trials are excluded when population firing rates are too low (<1 spk/s) to establish a stable firing estimate. Beginning-of-task (*t* = 1.1–1.2 s) and end-of-task (*t* = 2.9–3.0 s) kurtosis values were obtained by averaging all time points during those time periods. Comparisons of P1 kurtosis were assessed for significance by Kruskal–Wallis tests.

### 2.9. Spike timing data analyses

Phase-locking of neurons to the pulse stimuli was assessed by measuring the percentage of APs occurring during pulse presentations for each neuron. Regularity of spiking was assessed using coefficient of variation (CV). Synchrony of firing between pairs of neurons was quantified by measuring the percentage of coincident APs, normalized to the neuron with the higher overall firing rate. To facilitate comparison with phase-locking, APs were deemed coincident if they occurred within one pulse phase (300 *μs*) of each other. Differences in spike timing were assessed for significance by 1-way ANOVA.

## 3. Results

### 3.1. Experimental design

In this *in-silico* study, we assessed the ability of PS and direct current (a.k.a. ‘galvanic’) stimulation (GS) to bias large networks of neurons by exposing a well-established computational model of perceptual decision making [2] to both paradigms. In brief, this winner-take-all model consisted of two subpopulations of pyramidal neurons (P1 and P2) responsive to leftward and rightward dot motion respectively, NS pyramidal neurons, and a common pool of inhibitory interneurons (Int), shown in figure 1(A). All neurons were simulated with LIF dynamics and connected with varying synaptic weights as in Wang [2]. We used the neural network parameters described in that publication to build our network. Neurons were given strong connections $\left(w_{\text{strong}}=1.7\right)$ within subpopulations (ex. P1–P1), weak connections $\left(w_{\text{weak}}=0.8765\right)$ across subpopulations (ex. P1–P2), and medium-strength connections $\left(w_{\text{medium}}=1\right)$ with interneurons. In each of 100 trials, all neurons received 2400 Hz background Poisson-random excitatory input(AMPAEPSCs)resulting in spontaneous firing rates of 0–4 spk/s for pyramidal neurons and 5–7 spk/s for interneurons (figure 1(B)). During the experiment, from *t* = 1–3 s, P1 and P2 received differential task input (AMPA EPSCs) varying from 0–80 Hz depending on the task coherence for that trial (see Methods for details). For the example in figure 1, when the task coherence is −51.2%, P2 neurons receive task input EPSCs at 60 Hz and P1 neurons receive task input EPSCs at 20 Hz. For this control experiment, this was the only input to the model. As a result of this input, P1 and P2 firing rates increased until P2 exceeded a natural threshold of about 15 spk/s, and subsequently P2 firing rates rapidly increased, winning the decision. Subsequently, P1 firing rates were suppressed by feedforward inhibition from the interneurons (figure 1(B)). Consistent with previous work, we defined ‘winning’ and ‘losing’ by comparing the average firing rates of the pyramidal populations P1 and P2 at the end of each trial (see methods for details). If, during this period (*t* = 1–3 s) we exposed neurons in P1 to electrical stimulation, we could bias the network behavior, reversing the decision outcome, making P1 win and suppressing the P2 response (figure 1(C)). After task input and electrical stimulation were turned off, the high-firing-rate attractor state was self-perpetuated by the network in both control and stimulation trials as seen between 3 and 4 s in figures 3(B) and (C), respectively.

### 3.2. Electrical stimulation alters decision making

The electrical stimulation paradigms were point-source monopolar (referenced to distant ground) pulsatile charge balanced biphasic pulse trains (PS), and cathodic (excitatory) and anodic (inhibitory) galvanic currents (CGS and AGS respectively). The stimulation paradigms were assumed to create spherical electric fields in a homogeneous environment and implemented to affect pyramidal neurons within the LIF model based on their distance from the electrode as discussed in the Methods. P1 neurons were uniformly distributed around the electrode from 10 *μ*m–2 mm away based on staining work by Levitt *et al* [27], and PS pulse parameters by design matched those of Hanks *et al* [34]: 10 *μ*A, 300 *μ*s/phase, 200pulses s^−1^.

**Figure 2.**
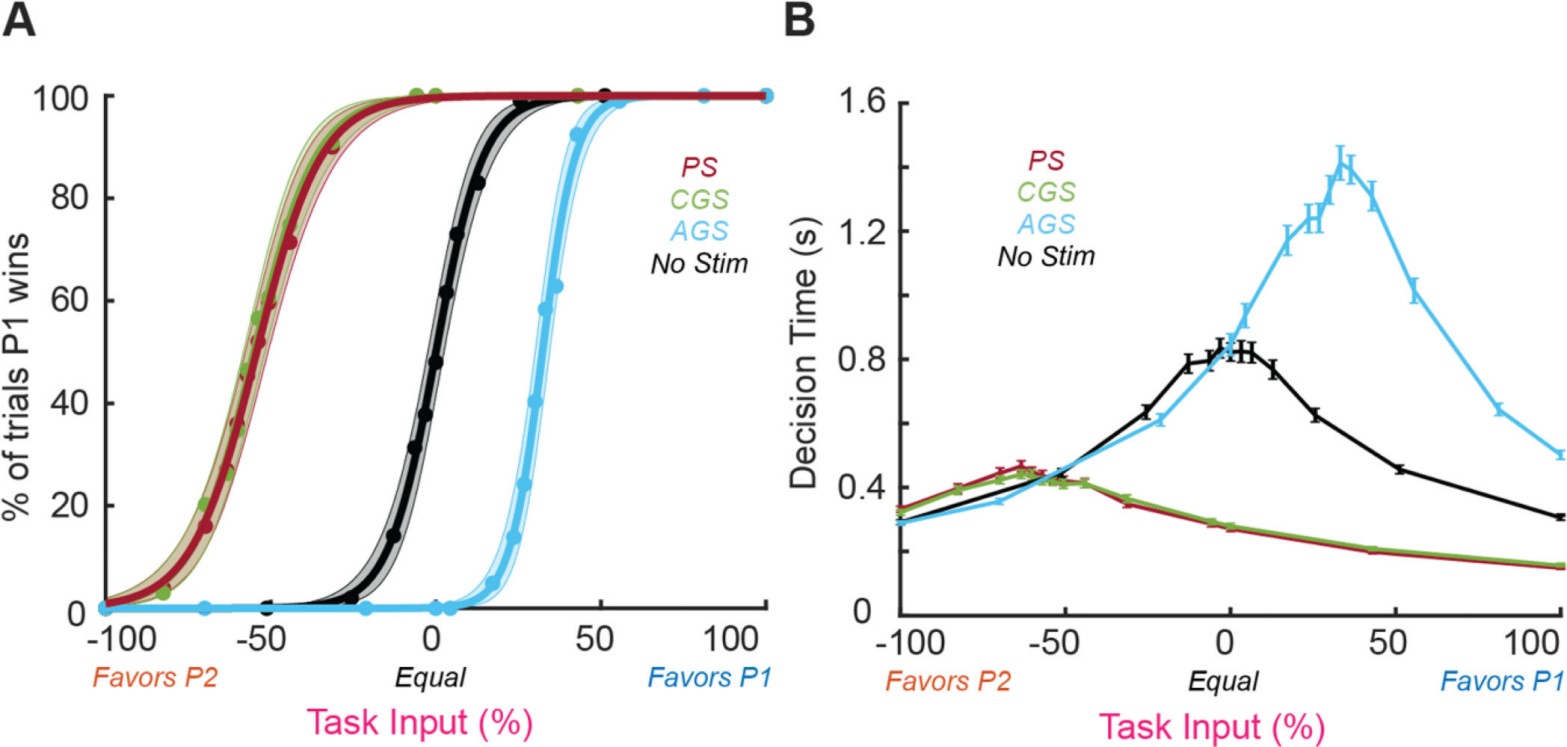
Effects of electrical stimulation on decision making and decision time (N=100 trials). The decision metrics are shown for pulsatile (red) and cathodic galvanic (green), anodic galvanic (blue), and no stimulation (black). (A) The percentage of trials in which the stimulated population (P1) wins the decision-making process are displayed for various levels of visual coherence. Psychometric curves are shown in bold with shaded regions indicating 95% bootstrapped confidence intervals (N=10 000 bootstraps). Red and green curves were calibrated to coincide by design to allow us to compare the subsequent effects of PS and CGS on the neural populations. (B) The time it takes for the winning population to clear the decision threshold (defined to be 15 spk/s) is shown. Error bars depict trial mean and standard error at each coherence level.

In figure 2, the behavioral effects of PS, CGS, and AGS on decision making were assayed by the percentage of trials in which the stimulated population (P1) won and the decision time. In control trials without electrical stimulation, the decision-making network produced a characteristic psychometric curve with no significant bias (*p* = 0.98 by 1-sample non-parametric bootstrap N=10 000). The percentage of trials in which P1 wins depended on the coherence of the task input, with −100% favoring P2 and +100% favoring P1 (figure 2(A), black). (see supplementary material table S7 for the values of the task input coherence parameters).

To compare the relative effects of excitatory stimulation from PS to those induced by CGS, we calibrated the input from CGS such that the bias of the two stimuli were statistically equivalent. We did this by adjusting the current amplitude of CGS, until its psychometric curve matched that of PS (green and red respectively are equal by design in figure 2(A)), resulting in CGS = −1.4 *μ*A. We then adopted AGS = −CGS = +1.4 *μ*A so that we could make a direct comparison to network behavior based on these stimulation paradigms. (see Supplementary Material table S5 for the final values of the stimulation parameters).

Excitatory stimulation from PS and CGS caused the network to favor P1, shifting the psychometric curve by −54.3% ± 1.8% and −55.0% ± 1.7% respectively (figure 2(A); PS in red, CGS in green). Electrical inhibition from AGS caused the network to favor P2, shifting the psychometric curve by +32.3% ± 1.1% (figure 2(A), blue). Although CGS and AGS used identical amplitude current (1.4 *μ*A), AGS shifted the psychometric curve significantly less (*p* < 10^−4^ by 2-sample non-parametric bootstrap N=10 000). CGS and PS induced statistically equivalent shift (*p* = 0.76 by 2-sample non-parametric bootstrap). PS and CGS also broadened the psychometric curve (although only PS was statistically significant), decreasing the slope by 0.94 ± 0.24 and 0.83 ± 0.28 respectively relative to no-stimulation (*p* = 0.022, *p* = 0.059 by 2-sample non-parametric bootstrap N=10 000). AGS steepened the psychometric curve, increasing the slope by 1.56 ± 0.53 (*p* = 0.009 by 2-sample non-parametric bootstrap N=10 000).

All three stimulation paradigms also affected the decision times (figure 2(B)). Excitatory stimulation from PS and CGS decreased peak decision time by 0.53 ± 0.02 s and 0.58 ± 0.02 s respectively and shifted the peak decision time by −57.5% ± 1.7% and −57.0% ± 1.8% (figure 2(B); PS in red, CGS in green). Inhibitory influence from AGS increased peak decision time by 0.69 ± 0.04 s and shifted the coherence by +31.3% ± 1.3% (figure 2(B) blue). As with the psychometric curve, AGS shifted the coherence significantly less than CGS (*p* = 3.1 × 10^−23^ by unpaired t-test). However, AGS induced statistically equivalent change in peak decision time as CGS (*p* = 0.34 by unpaired t-test). As with the psychometric curves, CGS and PS induced statistically equivalent coherence shift of the peak decision time (*p* = 0.83 by unpaired t-test).

These changes in decision making elicited by PS in our model are consistent with those observed in behavioral studies [34], albeit with larger magnitude. Psychometric curves are shifted such that stronger task-related input is required to make decisions against the stimulated population (P1). Decision times are decreased when task-related input and stimulation both favor P1 (e.g. at +25% coherence) but increased when task-related input and stimulation battle over control of the network (e.g. at −60% coherence; figure 2(B), red). CGS showed similar interactions (figure 2(B), green), while the effects of AGS were smaller in magnitude and opposite in polarity (figure 2(B), blue). The decreased effectiveness of AGS relative to CGS was likely due to a floor effect, since neurons cannot decrease their firing rates below 0 spk/s from a baseline of only 0–4 spk/s. Importantly, both excitatory stimulation paradigms (PS and CGS) pushed P1 firing rates toward the decision threshold and thereby decreased overall decision times; whereas electrical inhibition (AGS) pulled P1 firing rates away from the decision threshold and thereby increased overall decision times. Importantly, AGS inhibited P1 decision times so much that a substantial fraction of trials did not reach the decision threshold before the task period ended at t=3s. This effect is consistent with experimental studies using tDCS to bias decision making [35]. Similarly, both excitatory stimulation paradigms (PS and CGS) reduced the slope of the psychometric curves, whereas electrical inhibition (AGS) steepened the slope. These results agree with findings by Salzman *et al* [36] that PS of area MT significantly flattened psychometric curves. This line of evidence suggests that CGS can effectively mimic PS in the context of perceptual decision making, at a relatively low amplitude (−1.4 *μ*A).

### 3.3. PS and GS induce different profiles of neuronal activation/deactivation

PS and GS affect single neurons differently. PS has a strong depolarizing effect on supra-threshold neurons, primarily causing affected neurons to evoke APs in response to each pulse. GS on the other hand, smoothly modulates extracellular potentials, making affected neurons more likely or less likely to fire APs in response to EPSPs. We hypothesized that the effects of PS on neurons will be less affected by other neural connections due to its strong ability to evoke spikes.

#### 3.3.1. Responses in a completely disconnected network

**Figure 3.**
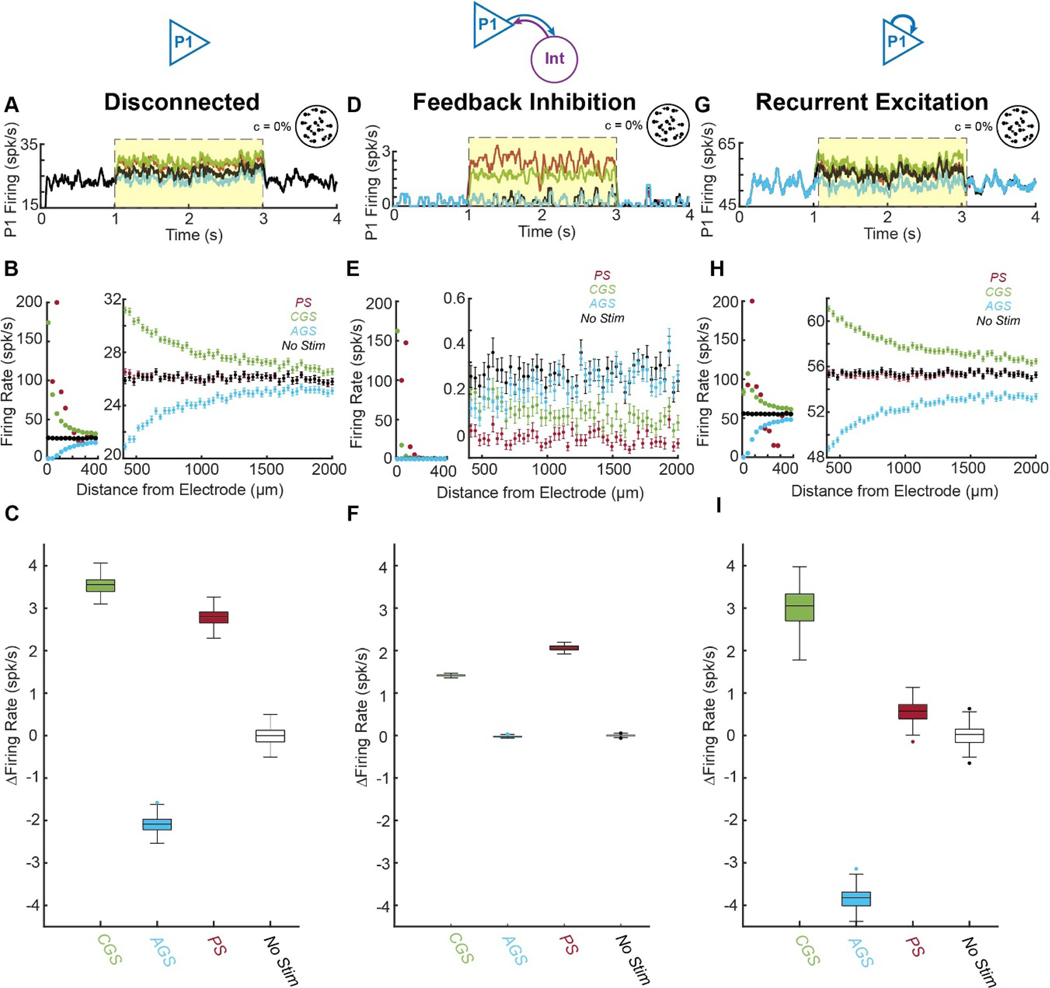
Effects of pulsatile (red), cathodic galvanic (green), anodic galvanic (blue), and control (black) stimulation on individual P1 neural firing rates (N=100 trials). Networks with fully disconnected neurons (A)–(C), feedback inhibition only (D)–(F), and recurrent excitation only (G)–(I) are investigated. Average P1 Firing for representative trials are shown in (A), (D) and (G). Each neuron’s mean task firing rate ±sem (*t* = 1–3 s) is shown as a function of its distance to the stimulation electrode during stimulation (yellow zone) (B), (E), (H); left: neurons <400 *μ*m from electrode, right; neurons >400 *μ*m from electrode). Box plots (C), (F), (I) depict each trial’s population-averaged change in firing rate relative to no stimulation. For all trials, task-related input was equal for P1 and P2 (coherence = 0%).

To investigate the mechanism of how PS and GS interact with the neural network, we first ascertained the effects of PS and GS in disconnected neurons, leveraging the benefits of the experiment being conducted entirely *in-silico*. Disconnecting the neurons in P1 allowed us to measure firing rates over the entire task period (*t* = 1–3 s) without the confounds induced by network activity (figure 3(A)).

As expected from single neuron studies, during stimulation (figure 3(A), yellow zone) the distributions of firing rates induced by PS and CGS were significantly different from each other and matched the expectations of single neural responses to stimulation (*p* = 1.1 × 10^−18^ by Kolmogorov–Smirnov test; figure 3(B)).

For PS, due to the refractory effects of high-amplitude pulses, the neurons closest to the PS electrode (<40 *μ*m) were blocked. Most of the neurons farther away (44–314 *μ*m) were excited with decreasing level of excitation as a function of distance. Excitation was limited to the neurons close to the PS electrode up to m from the stimulation site (347*μ* by unpaired 1-tailed t-test with Bonferroni correction; figure 3(B) red, right panel).

In contrast, for CGS, the neurons closest to the stimulation electrode (<400 *μ*m) were strongly excited with firing rates up to 173 spk/s (figure 3(B) green, left panel). In addition, weak excitation spread far from the GS electrode with small but significant increases in neural firing rates up to 1798 *μ*m away (*p* = 0.83 *p* < 0.05 by unpaired 1-tailed t-test with Bonferroni correction; figure 3(B) green, right).

Compared to CGS, AGS had equal and opposite effects on neural firing rates for neurons > 246*μm* away (*p* > 0.05 by unpaired 2-tailed t-test with Bonferroni correction; figure 3(B), blue). However, it completely blocked the activity of neurons <50 *μm* away and thereby induced a smaller change in firing rate compared to CGS (figure 3(B), blue). As a result, AGS induced a smaller average change in firing rate (median: 2.09 spk/s IQR: 1.97–2.22 spk/s) compared to CGS (median: 3.56s pk/s IQR: 3.39–3.67 spk/s) in the P1 population (*p* = 2.6 × 10^−34^ by Kruskal–Wallis test; figure 3(C)).

PS induced a smaller change in firing rate (median: 2.80 spk/s IQR: 2.65–2.91 spk/s) than CGS when averaged over the entire neural population of P1, likely due to its smaller spread of activation (*p* = 6.7 × 10^−9^ by Kruskal–Wallis test; figure 3(C)).

From these results in disconnected neurons, one would expect that CGS would induce a greater change in decision-making than PS since it affects more neurons and induces a greater overall change in firing rate. However, by design they induce identical effects in a fully connected network (figure 2(A)), suggesting that PS and GS interact with the interconnected network in more complicated ways.

#### 3.3.2. Network with only inhibitory feedback

We next probed the effects PS and GS in the context of feedback inhibition. We added 30 inhibitory interneurons connected to the P1 population to create the 80/20 *E/I* ratio, which yielded a stable P1 spiking activity during PS and CGS (figure 3(D), yellow zone).

Feedback inhibition dramatically reduced spontaneous firing rates from ∼25 spk/s to <1 spk/s. Nevertheless, some neurons closest to the CGS and PS electrodes (<100 *μ*m) were still strongly activated achieving firing rates up to 162 spk/s from CGS and 147 spk/s from PS (figure 3(E), left panel). However, the far-reaching weak excitation induced by CGS seen in the disconnected network (figure 3(B), right panel) was severely attenuated by compensatory feedback inhibition (figure 3(E), right panel). CGS only induced increases in neural firing rates up to 178 *μ*m away from the electrode (*p* < 0.05by unpaired 1-tailed t-test with Bonferroni correction; figure 3(E) green, right panel).

P1 neurons stimulated by PS also experienced compensatory feedback inhibition, causing 51 neurons 500–2000 *μ*m away from the electrode to have lower firing rates than non-stimulated control (*p* < 0.05by unpaired 1-tailed t-test with Bonferroni correction; figure 3(E) red, right panel).

As a result, both CGS and PS were significantly less effective at increasing neural firing rates under feedback inhibition than with disconnected neurons (*p* = 2.6 × 10^−34^ and *p* = 2.6 × 10^−34^ respectively by Wilcoxon rank sum test; figure 3(C)&F). Importantly, however, CGS induced a smaller average increase in firing rate (median: 1.41 spk/s IQR: 1.40–1.43) than PS (median: 2.06 spk/s IQR: 2.02–2.11 spk/s) under feedback inhibition (*p* = 5.6 × 10^−9^ by Kruskal–Wallis test), indicating that the effects of PS are more resistant to network-based suppression.

The effects of AGS were almost completely indistinguishable from the non-stimulated control under strong feedback inhibition (figures 3(E) and (F)), providing further support for the hypothesis that the limited effectiveness of AGS is driven by a floor effect.

From these results in neurons exposed to feedback inhibition only, one would expect that PS would induce a greater change in decision making than CGS since it induces a greater increase in average firing rate. On the contrary, we observed an equivalent effect, indicating that another network effect may be influencing decisions.

#### 3.3.3. Network with only excitatory feedback

Finally, we investigated the effects of GS and PS with recurrently connected P1 neurons only. To avoid runaway recurrent excitation, we added *weak* recurrent excitatory connections (*w*_rec_ = 0.05) among the neurons in P1 and still maintained a stationary process (figure 3(G)). Recurrent excitation dramatically increased spontaneous firing rates from ∼25 spk/s to ∼52 spk/s.

Despite the increased baseline firing rates, the neurons exposed to PS experienced a similar activation profile as in the disconnected case with a few neurons firing ∼90 spk/s, one neuron fully activated at 200 spk/s, and neurons >145 *μ*m away not significantly increased relative to control (*p* < 0.05 by unpaired 1-tailed t-test with Bonferroni correction; figure 3(H), red).

In contrast, CGS activation scaled synergistically with the recurrent excitation, inducing higher firing rates than in the disconnected case for all but the closest neuron (figure 3(H), green; compared to figure 3(B), green). Due to the high excitatory current from both CGS and recurrent inputs, the closest neuron experienced intermittent depolarizing block, which caused its average firing rate to decrease to 82 ± 7 spk/s, which was highly variable among trials. Importantly, CGS induced a significantly greater increase in firing rate than PS under recurrent excitation (*p* = 7.6 × 10^−10^ by Kruskal–Wallis test), indicating that the effects of CGS are more synergistic with network-based excitation.

Under recurrent excitation, AGS induced a larger change in firing rates (median: 3.82 spk/s IQR: 3.69–4.01 spk/s) than in the disconnected case (median: 2.09 spk/s IQR: 1.97–2.22 spk/s) due to the elevated spontaneous firing rates and removed floor effect (*p* = 2.6 × 10^−34^ by Kruskal–Wallis test; figure 3(C)&I, blue).

Since membrane voltage changes in response to suprathreshold pulses are not affected by small fluctuations in membrane potential, neurons that are directly affected by PS are not very sensitive to either feedback inhibition or recurrent excitation. In contrast, GS induces small changes in membrane potential that increase (for CGS) or decrease (for AGS) the probability of firing APs in response to naturally occurring EPSCs and IPSCs [29]. For this reason, CGS and AGS are more sensitive to the ongoing network activity, and significantly alter average firing rates farther from the site of the electrode than PS. As a result of these phenomena, increasing the strength of feedback inhibition in the network gives PS more influence on the network activity relative to GS; whereas increasing the strength of recurrent excitation in the network gives GS more influence over network activity relative to PS.

### 3.4. PS vs. GS effectiveness depends on dynamic E/I balance changes

Based on the results obtained with modified excitation/inhibition in the previous section, we hypothesized that the ability of GS to modulate the fully connected network activity would be highly dependent on the excitatory/inhibitory balance. In contrast, the ability of PS to evoke spikes should largely remain unchanged independent of this network behavior.

**Figure 4.**
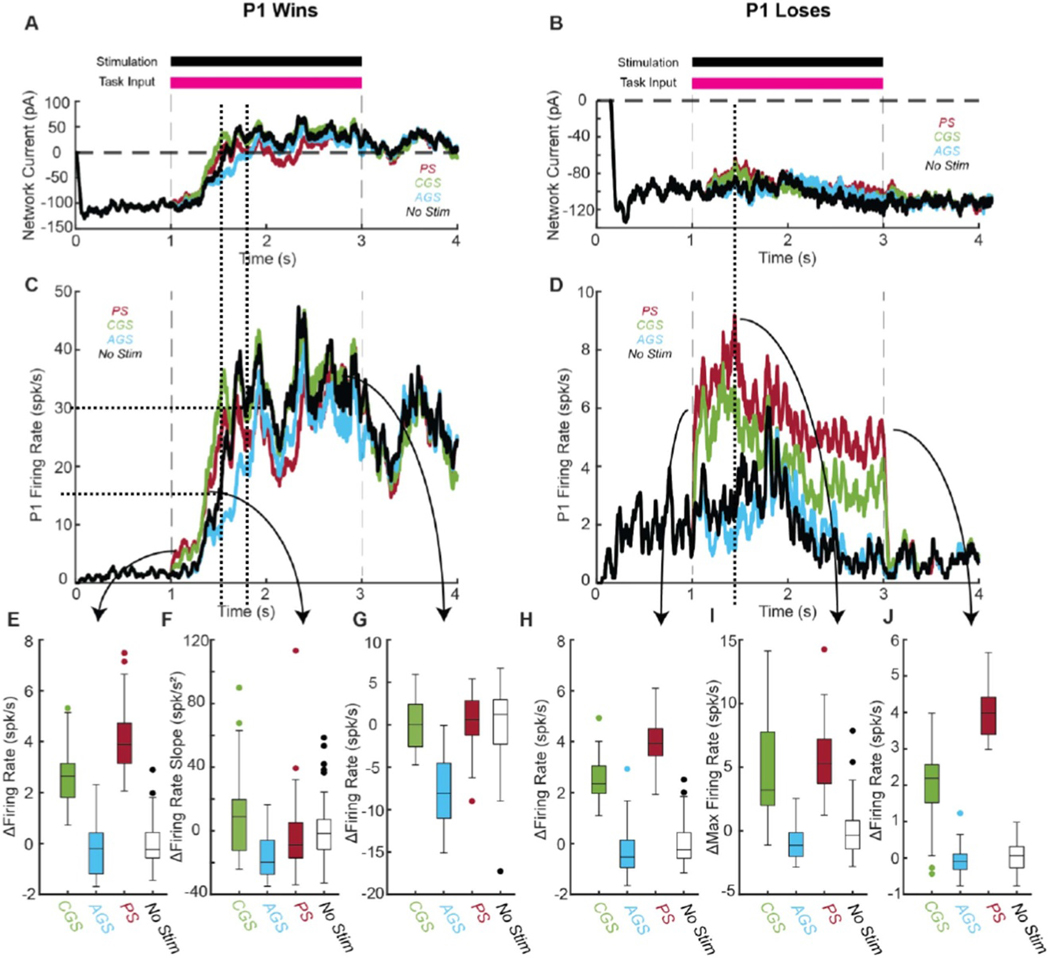
Effects of pulsatile (red), cathodic galvanic (green), anodic galvanic (blue), and control (black) stimulation on P1 population firing rates over time (N=100 trials). Trials in which P1 won (left, (A), (C), (E)–(G) and P1 lost (right, (B), (D), (H)–(J)) are investigated separately. (A), (B) Network currents $\left(I_{{\text{AMPA}}_{\text{rec}}}+I_{\text{NMDA}}-I_{\text{GABA}}\right)$ received by P1 neurons are shown in two representative trials. (C), (D) P1 firing rate trajectories are shown over time in two representative trials. (E), (H) Box plots depict the population-averaged change in firing rate relative to no stimulation for the first 100 ms of the trial (*t* = 1–1.1 s). (F) Box plots depict the population-averaged change in firing rate slope around the decision threshold of 15 spk/s relative to No Stim. (I) Box plots depict the population-averaged maximum firing rate relative to No Stim. (G), (J) Box plots depict the population-averaged change in firing rate relative to No Stim for the last 100 ms of the trial (*t* = 2.9–3 s). For all trials, task-related input was set such that P1 and P2 each won 50% of the trials (coherence = 0% for No Stim, −57% for Pulsatile and Cathodic Galvanic, and +30% for Anodic Galvanic). Only trials in which P1 won before *t* = 2.5 s were included in the analysis.

Because of the recurrent nature of the network, the relative strengths of feedback inhibition and recurrent excitation change during the time course of each trial, with or without electrical stimulation. We measured the relative impact of these two network motifs by recording the recurrent AMPA, NMDA, and GABA currents experienced by each neuron in P1. We indexed the overall *network current* by adding the recurrent excitatory AMPA and NMDA currents and subtracting the inhibitory GABA current. Thus, when the *network current* is negative, the network favors feedback inhibition, but when it becomes positive it favors recurrent excitation (figure 4(A) and (B)).

At baseline, feedback inhibition is substantially stronger than recurrent excitation (≈−100 pA), which holds the pyramidal neuron firing rates at 0–4 spk/s (figures 4(A)–(D)). Once the task-related input turns on at *t* = 1 s, input excitation and recurrent excitation together overpower feedback inhibitionand pyramidal firing rates begin to rise. As P1 firing rates rise, recurrent excitation grows, creating a positive feedback loop. If P1 wins, when average P1 firing rates clear the critical decision threshold of 15 spk/s, this positive feedback loop overwhelms feedback inhibition, and P1 firing rates surge rapidly up to ∼30 spk/s. In this elevated firing rate state, recurrent excitation is dominant (+25–50 pA) and sustains high firing rates (30–40 spk/s) even after the task-related input turns off at t=3s (figures 4(A) and (C)) dotted lines identify time indices for 15 and 30 spk/s). If P1 loses, it is instead strongly suppressed by feedforward inhibition after P2 clears the decision-making threshold. In this case, feedback inhibition is dominant throughout the trial, but recurrent excitation is most competitive (≈ −70*pA*) just before P2 clears the decision-making threshold, when both populations’ firing rates are elevated (∼10 spk/s) (figure 4(B) and (D), dotted line). Therefore, the relative strengths of feedback inhibition and recurrent excitation depend on both the outcome (P1 winning or losing) and time course of each trial.

Based on the results in the simplified networks, we hypothesized that PS would become more effective as feedback inhibition became more dominant, and CGS would become more effective as recurrent excitation became more dominant in the network. These differences should be apparent in the firing rate trajectories of winning and losing trials. Indeed, we observed distinct differences in the average P1 firing rate trajectories between PS, CGS, and AGS (figures 4(C)–(J)). These differences occurred even though PS and CGS induced equivalent changes in decision making.

We first assessed the effectiveness of PS and GS at the beginning of each trial to understand their immediate effects on the decision-making network (figures 4(E) and (H)). In the first 100 ms of the task period (*t* = 1–1.1 s) PS caused a larger increase in P1 firing rates (median: 3.89 spk/s IQR: 3.14–4.73 spk/s) compared to CGS (median: 2.64 spk/s IQR: 1.81–3.14 spk/s) in trials in which P1 won (figure 4(E); *p* = 0.078 by Kruskal–Wallis test). PS also caused a significantly larger increase in P1 firing rates (median: 3.93 spk/s IQR: 3.45–4.51 spk/s) than CGS (median: 2.34 spk/s IQR: 1.97–3.05 spk/s) in the first 100 ms (*t* = 1–1.1 s) of trials in which P1 lost (figure 4(H); *p* = 6.9 × 10^−3^ by Kruskal–Wallis test). This result is consistent with the hypothesis that PS is more effective than CGS when feedback inhibition is strongly dominant in the network (≈ −100 pA at 1.1 s in figures 4(A) and (B)). This finding is independent of the outcome of the trial because feedback inhibition is dominant at the beginning of both winning and losing trials.

Next, we assessed the effectiveness of PS and GS Next we assessed the effectiveness of PS and GS around the decision-making threshold (figures 4(F) and (I)) to understand how their effects integrate with dynamic network activity.

In winning trials, we observed that the slope of the P1 firing rate curve around the decision threshold (15 spk/s) was significantly affected by both PS and CGS (figure 4(F); *p* = 4.2 × 10^−5^ by Kruskal–Wallis test). The slope of the firing rate was increased by CGS (median: 8.72 spk/s^∧^2 IQR: −12.38–19.63 spk/s^∧^2), but decreased by PS (median: −8.89 spk/s^∧^2 IQR: −17.01–5.08 spk/s^∧^2) and AGS (median: −19.82 spk/s^∧^2 IQR: −27.31-(−6.00) spk/s^∧^2) compared to No Stim (figure 4(F)). As a result, CGS had a significantly higher median slope than PS (*p* = 0.03 by Kruskal–Wallis test) and AGS (*p* = 0.005 by Kruskal–Wallis test). This finding is consistent with the hypothesis that CGS is synergistic with recurrent excitation, since recurrent excitation drives the rapid increase in P1 firing rates around the decision threshold of 15 spk/s. On the other hand, PS-induced activation does not synergize well with recurrent excitation, limiting its effectiveness to rapidly increase firing rates around the decision threshold. The inhibitory effectiveness of AGS is increased as P1 firing rates increase due to the relaxing of the floor effect, which also results in a shallower slope.

In losing trials, we observed that the maximum P1 firing rate was increased by PS (median: 5.30 spk/s, IQR: 3.72–7.20 spk/s) and CGS (median: 3.21, IQR: 1.99–7.78 spk/s), but decreased by AGS (median: −1.13 spk/s, IQR: −2.01-(−0.14) spk/s) relative to No Stim (figures 4(D) and (I)). PS and CGS induced statistically equivalent increases in maximum P1 firing rate (*p* = 0.44by Kruskal–Wallis test). Our explanation for these equivalent increases in firing rate is that CGS becomes more effective relative to PS with even a slight increase in recurrent excitation (figure 4(D), dotted line).

Finally, we assessed the effectiveness of PS and GS at the end of each trial to understand their long-term effects on the network in steady state (figures 4(G) and (J)). In the last 100 ms of the task period (t = 2.9–3 s), average P1 firing rates were increased by PS (median: 0.58 spk/s IQR: −1.25–+ 2.83s pk/s), not affected by CGS (median: 0.00 spk/s IQR: −2.59–+ 2.41 spk/s), and decreased by AGS (median: −8.09 spk/s IQR: (−11.02)–(−4.52)) relative to No Stim in trials in which P1 won (figure 4(G)). Crucially, PS and CGS induced statistically equivalent changes in P1 firing rates relative to No Stim (*p* = 0.82 by Kruskal–Wallis test). This finding was somewhat surprising, and not immediately consistent with our hypothesis. Recurrent excitation is strongly dominant at the ends of trials in which P1 wins (+25–50 pA at 3 s; figure 4(A)), so we expected CGS to induce a greater increase in P1 firing rates than PS. Instead, we observed that CGS was equivalent to and even slightly less effective than PS. Upon further investigation, we found that shortly after P1 wins, the dramatic increase in recurrent excitation caused one of the P1 neurons to experience depolarizing block from CGS. This outlier drastically reduced average P1 firing rates, while not affecting the decision-making outcome of the trial, since the decision threshold had already been cleared.

In the last 100 ms of losing trials (*t* = 2.9–3 s), P1 firing rates were increased by PS (median: 3.98 spk/s IQR: 3.40–4.42 spk/s) and CGS (median: 2.19spk/s IQR: 1.52–2.56 spk/s) but decreased by AGS (median: −0.10 spk/s IQR: −0.31–(+0.11) spk/s) relative to no stimulation (figure 4(J)). As expected, PS caused a significantly larger increase in P1 firing rates than CGS (*p* = 3.8 × 10^−4^ by Kruskal–Wallis test). These findings are consistent with the hypothesis that increased feedback inhibition favors PS relative to CGS. At the ends of trials in which P1 loses, feedback inhibition is as dominant as it gets (−100–(−120)pA), and the difference between PS and CGS is also at its maximum (∼2 spk/s). Additionally, AGS is much more effective in winning trials than losing trials due to the relief of the floor effect. These differences in P1 firing rate trajectories over the time course of each trial represent measurable predictions about the different effects of PS and GS on functional networks of neurons that arise directly from a mechanistic understanding of their differing responses to network motifs of excitation and inhibition.

### 3.5. PS and GS induce different spatiotemporal distributions of activation

**Figure 5.**
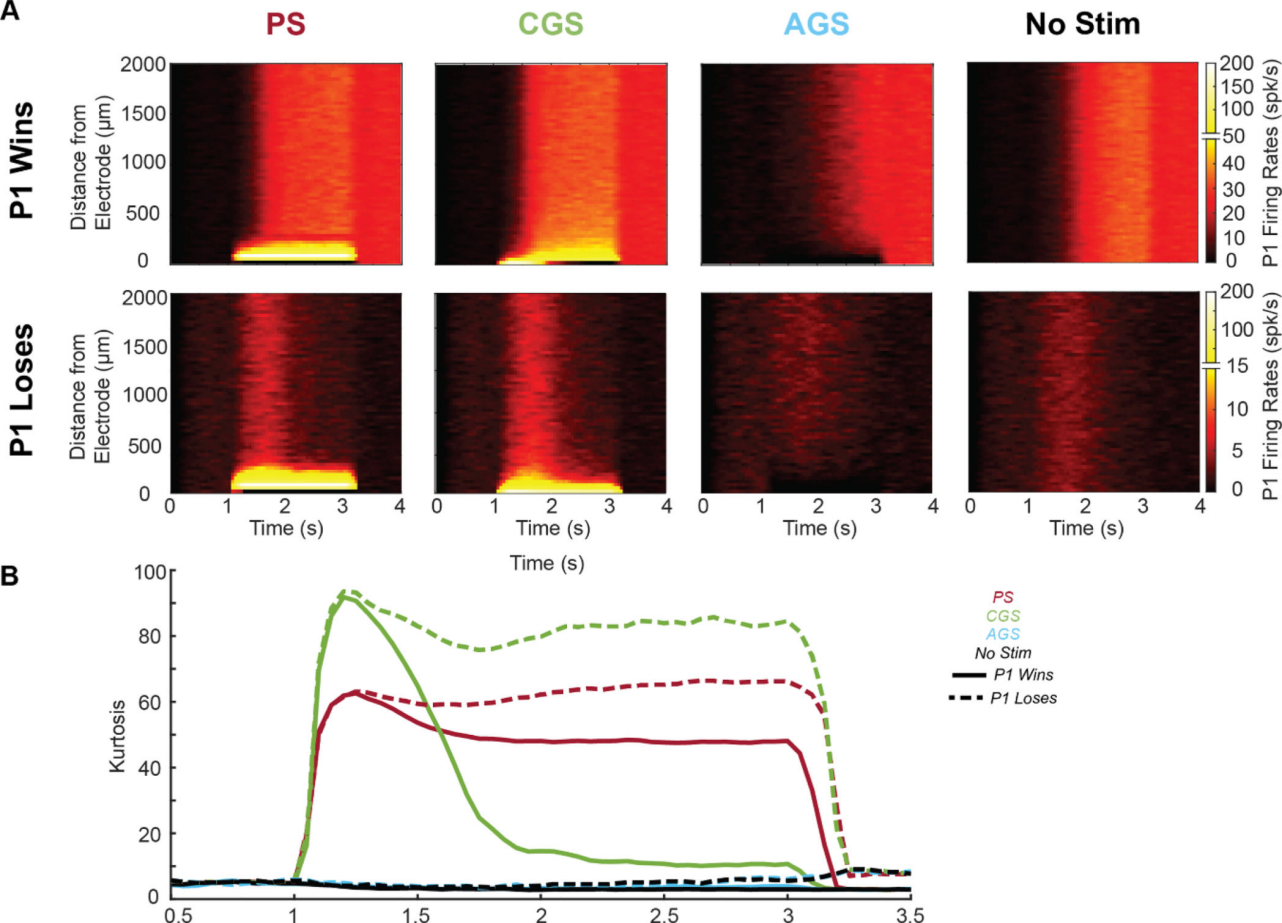
Effects of pulsatile (red), cathodic galvanic (green), anodic galvanic (blue), and control (black) stimulation on distributions of P1 firing rates over time (N=100 trials). (A) Heatmaps show individual neural firing rates for trials in which P1won (top) trials in which P1 lost (bottom). (B) Kurtosis of the distribution of P1 firing rates is shown over time. Trials in which P1 won are represented by solid lines, and trials in which P1 lost are shown by dashed lines. Beginning (0–0.5 s) and end (3.5–4 s) of trials are excluded due to artifactual effects when population firing rates are too low (<1 spk/s). For all trials, task-related input was set such that P1 and P2 each won 50% of the trials (coherence = 0% for No Stim, −57% for Pulsatile and Cathodic Galvanic, and +30% for Anodic Galvanic). Only trials in which P1 won or lost before *t* = 3 s were included.

Because PS appears to be generally less susceptible to network effects, we expect to find high variability in neural responses as we examine the PS effect on neurons at different distances from the electrode. PS should affect neurons nearby more than neurons farther away. In contrast, we would expect the effect of GS to be more uniform and less dependent on distance. To examine these hypotheses, we investigated the distribution of neural firing rates in the fully connected network. We visualized the neural activity as it evolved during the time course of each trial and as a function of distance from the electrode. To quantify the differences in variability of neural firing rates across the population of neurons, we used the fourth central moment: kurtosis as a measure of variability of firing rates across the neural population (figure 5(B)). High kurtosis values indicate high variability and low kurtosis values indicate uniformity of firing rates across population. We selected this metric due to its ability to highlight the most dramatic changes in neural firing rates such as blocking (<1 spk/s) and hyper-excitation (>100 spk/s).

As expected from the disconnected trials, CGS and PS strongly excited a small subset of neurons close to the electrode (<500 *μ*m) up to 200 spk/s (figure 5(A)). PS also induced full pulse-pulse block-ing in the closest neuron. These strong local effects are seen as increases in kurtosis at the beginning of the task period (*t* = 1.1–1.2 s) of both winning and losing trials (figure 5(B)). During this onset, CGS strongly activated only the closest neuron, whereas PS activated a larger block of close by neurons. As a result, CGS induced significantly larger kurtosis transients (median: 82.87 IQR: 76.63–87.83) compared to PS (median: 57.31 IQR: 53.51–60.48 *p* = 2.6 × 10^−5^ by Kruskal-Wallis test).

In winning trials, PS caused relatively static excitation (figure 5(B), solid red), but CGS excitation changed with time (figure 5(B), solid green). As P1 firing rates increased, CGS activation spread due to the increased recurrent excitation, captured by the falling kurtosis levels. PS activation also spread, but much less than CGS. As a result, by the end of the task period (*t* = 2.9–3 s) of winning trials, PS maintained the large nonuniformity in firing rates, shown by the significantly higher kurtosis (median: 47.88 IQR: 46.56–49.53) than CGS (median: 10.48 IQR: 9.19–11.51 *p* = 2.4 × 10^−5^).

In losing trials, P1 firing rates achieved a maximum around 1.5 s before P2 cleared the decision-making threshold, after which P1 was suppressed. Accordingly, CGS achieved its maximal spread throughout the network during this time, and then reverted to strongly activating only a few neurons (figure 5(A), bottom). As a result, similar to the initial transients, CGS maintained higher (but not significantly higher) kurtosis (median: 101.61 IQR: 48.96–106.15; figure 5(B), dashed green) than PS (median: 66.43 IQR: 64.70–68.71; figure 5(B), dashed red) at the end of the task period (*t* = 2.9–3 s) of trials in which P1 lost (*p* = 0.22 by Kruskal–Wallis test).

AGS induced a mild deactivation, especially prominent for the closest neurons (<500 *μ*m) in winning trials. AGS did not affect firing rates dramatically in losing trials due to the floor effect. As a result, AGS did not induce significant changes in firing rate distributions compared to No Stim (*p* > 0.05 by Kruskal–Wallis test for all comparisons).

These findings support the hypothesis that CGS induces a more uniform spread of activation that is highly network-dependent, whereas PS activates a single block of neurons relatively statically throughout the task period. Interestingly, at the beginning of the task period, when P1 firing rates are low, CGS induced higher kurtosis than PS (figure 5(B)). This indicates that CGS takes some time for its effects to spread throughout the network, and initially strongly activates fewer neurons than PS. Similarly, when P1 lost and firing rates remained low, CGS induced higher kurtosis than PS, suggesting that high levels of recurrent excitation relative to feedback inhibition are necessary to propagate the effects of CGS throughout the network. This finding is consistent with the results from the simplified networks, in which feedback inhibition attenuated the spread of activation induced by CGS.

### 3.6. PS but not GS induces synchronous firing in the closest neurons

The decision-making model employed here assumes that spatiotemporal integration of neural firing exclusively determines the perceptual decision-making process. However, recent evidence suggests that precise spike timing may play a complementary role in determining when to prioritize certain streams of information over others [37, 38]. Given that PS is more likely to influence neurons independent of network activity, we expect that the effect of PS on neural firing would be stronger and more phase-locked at the start of the stimulation, while the effect of GS on the population would be more distributed and less correlated.

**Figure 6.**
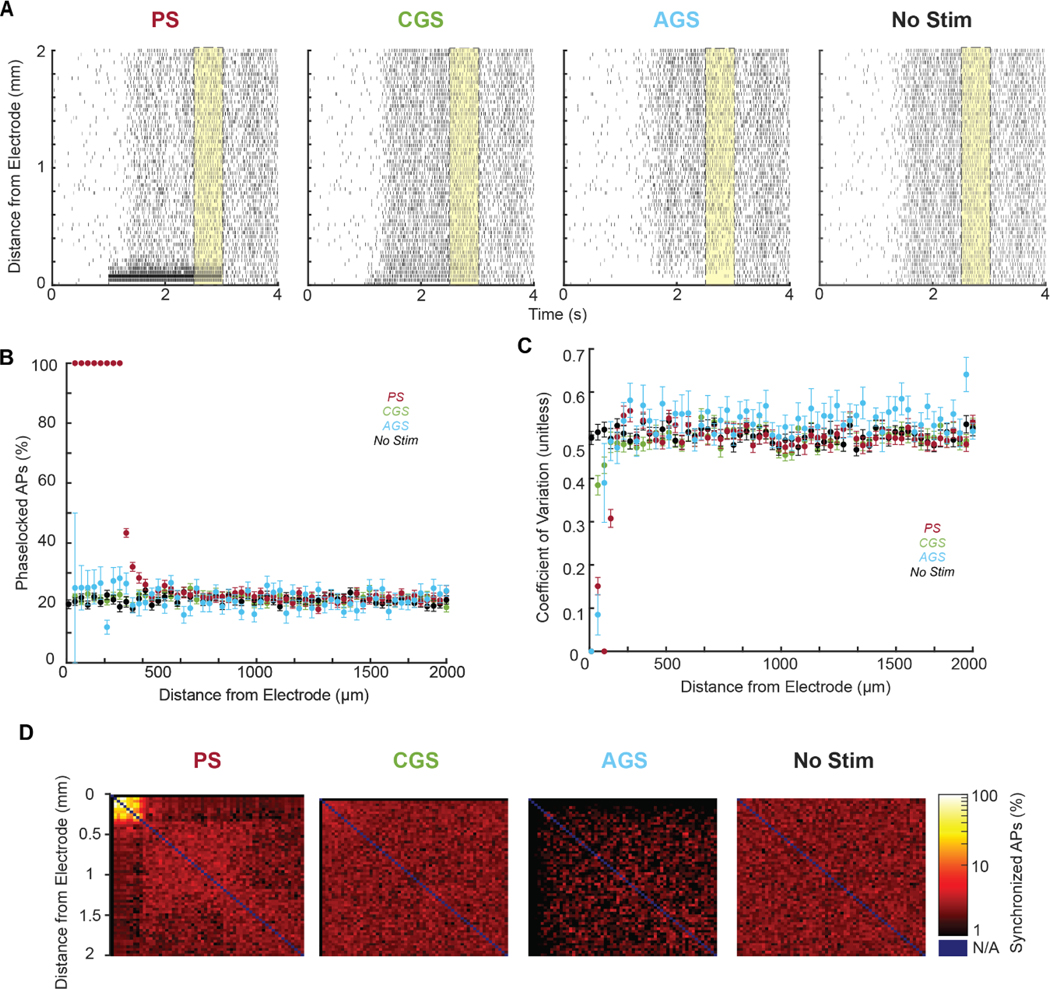
Spike timing differences in P1 neurons among stimulation conditions (Pulsatile, red; cathodic galvanic, green; anodic galvanic, blue; and no stimulation, black) for N=100 trials. (A) Representative trial rasters are plotted for each stimulation condition, with end-of-task period (*t* = 2.5–3 s) highlighted in yellow. (B) The percent of each neuron’s APs that occur during a pulse presentation for the end-of-task period is shown as a function of its distance to the stimulation electrode. (C) Each neuron’s end-of-task coefficient of variation (CV) is shown as a function of its distance to the stimulation electrode. (D) Heat map of the percent of APs from a neuron that are synchronized to another neuron as a function of distance from the stimulation electrode. Self-synchrony was undefined (*N*/*A*, blue). For all trials, task-related input was set such that P1 and P2 each won 50% of the trials (coherence = 0% for no stim, −57% for pulsatile and cathodic galvanic, and +30% for anodic galvanic). Only trials in which P1 won before *t* = 3 s were included.

To understand the steady-state effects of each stimulation modality, we measured spike timing in the last 0.5 s of the task period (*t* = 2.5–3 s; figure 6(A), yellow). We found that PS induced fully phase-locked activity in the responding neurons closest to the electrode (<300 *μ*m), and partially phase-locked activity (20%–60% phase-locked aPs) in neurons a moderate distance away (300–400 *μ*m) in P1 (figure 6(B), red). For each neuron in P1, we computed the CV to measure regularity of firing. PS induced highly regular firing (0–0.4) in a small number of neurons closest to the electrode (<150 *μ*m); whereas CGS and AGS caused a more modest increase in regularity (figure 6(C)). Last, for each neuron pair in P1, we calculated the percentage of synchronized aPs (<300 *μ*s apart) in figure 6(D). We observed a large increase in synchrony among the neurons phase-locked to pulses (up to 100% synchronized <300 *μ*m from the electrode; figure 6(D), left). These neurons were the primary drivers of an overall increase in synchrony from PS (*p* < 10^−15^ by 1-way ANOVA). In contrast, CGS did not induce significant synchrony compared to control (figure 6(D), middle-left; *p* = 0.930.93 by 1-way ANOVA). AGS caused a mild desynchronizing effect (figure 6(D), middle-right; *p* < 10^−15^ by 1-way ANOVA).

From these results, we conclude that PS induces phase-locked aPs in the directly activated population, which manifest synchronized connections in that subset of neurons. CGS largely preserves spike timing and AGS induces a mild desynchronization. Interestingly, based on the CV statistic, these neurons are not firing as regularly as one might expect. This is likely due to neurons phase-locking to an irregular subset of pulses based on variable relative refractory periods.

## 4. Discussion

By design, both CGS and PS achieved the same strong biasing effect and decreased decision time. Given this equivalence, the two stimulation methodologies exhibited nuanced differences in the ways that they interacted with the decision-making network to achieve this bias. PS elicited responses within the network by strongly and synchronously activating a static block of neurons close to the electrode during both winning and losing trials. In contrast, CGS directly activated a much smaller number of neurons, which yielded a smaller increase in firing rates at the task onset. As the task progressed, the synergy between CGS and recurrent excitation led to faster accumulation of perceptual evidence and a steeper slope in the transition between low P1 firing and high P1 firing. This synergy also contributed to a broader spread of activation in winning trials. When P1 lost, CGS’s sensitivity to feedback inhibition allowed P1 to be appropriately suppressed, but with some unnatural residual activation. Throughout winning trials, CGS did not induce any additional synchrony, generally respecting the spike timing transmitted from the EPSCs/IPSCs analogous to that of the non-stimulated trials. In general, these results show that CGS produces more spatiotemporally distributed responses primarily due to its synergistic relationship with recurrent excitation; whereas PS produces more synchronized responses due to the phase-locked nature of its AP initiation.

One limitation of this study is that the effect of PS on decision making in the model is larger than the effect *in vivo* with the same stimulation parameters [34]. Similar studies with lower pulse amplitude (5 *μ*A) were shown to achieve an equivalent effect on decision making in some experiments, also highlighting the apparent inter-subject variability [7, 39]. Another limitation is that we only considered electrical activation of excitatory pyramidal neurons in our model, even though inhibitory neurons are co-located in the decision-making circuit. Inhibitory neurons have much smaller axons, so they experience a much weaker effect of electrical stimulation. Also, similar modeling studies suggest that the effects of electrical stimulation on decision-making are primarily driven by its effect on excitatory pyramidal neurons rather than inhibitory interneurons [35]. Nevertheless, there may be some nuanced effect via the interneurons that would be interesting to explore in future work.

We assessed one pair of PS and GS amplitudes, each of which gives rise to a specific pattern of activation/deactivation depending on distance from the electrode. We chose this approach to enable direct comparison to existing experimental work in the literature [34]. CGS required a nearly 10-fold lower amplitude at 1.4 *μ*A to achieve an equivalent behavioral effect as PS at 10 *μ*A. This is consistent with the standard strength-duration curve that attributes lower thresholds for long duration stimuli due to the prolonged time given to charge the membrane capacitance and consequently induce larger effects on the membrane voltage.

To compare PS to GS in this study we specifically chose PS train parameters to mimic the stimulation paradigm of the previous experiments [34]. Previously published experimental work as well as our own computational analysis suggests that varying pulse rate has strong nonlinear effects on the resulting firing rate of targeted neurons especially for high amplitude pulses and in the presence of spontaneous activity [28–30]. Indeed, in this publication we see this effect with the inhibition of the neurons closest to the electrode that experience the highest amplitude. For this reason, to achieve more predictable results using PS, we would expect that small amplitude stimuli would be more effective over a wider range of pulse rates than using large amplitude pulses, assuming that the recruitment of the neural population is acceptable within the activated network.

Based on the modeling experiments in this work, GS appears able to interact with functional neural circuits as effectively as PS, while minimizing undesired side-effects such as excessive neural synchrony, uneven distributions of neural activation, and insensitivity to ongoing network dynamics. These effects were largely consistent with our hypotheses based on previous modeling work in single neurons but differed in small ways idiosyncratic to the stimulation protocol we used. Specifically, our CGS amplitude was sufficient to directly activate a few neurons closest to the electrode regardless of EPSCs/IPSCs. These neurons fired more regularly (as measured by CV), remained somewhat active under feedback inhibition, and one neuron experienced depolarizing block under sufficient recurrent excitation. This small minority differed from the overall trends of activation and was responsible for unexpected findings such as the decrease in efficacy at the ends of trials in which P1 won. Future work exploring different parameter combinations of PS and CGS will be instrumental in determining whether these depolarizing block effects consistently arise or depend on specific CGS amplitudes.

In addition to excitation, GS can readily support electrical inhibition via AGS. Inhibiting P1 neurons via AGS caused a behavioral bias opposite in polarity, but smaller in magnitude than the bias induced by CGS, due to the floor effect on firing rates. Inhibitory AGS also increased decision times and psychometric sensitivity, whereas excitatory CGS and PS decreased decision times and psychometric sensitivity. These findings, together with related work [3, 35, 36], paint a picture in which excitation drives fast, imprecise decisions, and inhibition drives slow, precise decisions. Despite its substantial behavioral bias, AGS did not induce any alterations in firing rate distributions, and it avoids the risks of excessive neural activation such as excitotoxic shock. Therefore, AGS may be an even better candidate for effective neural interfacing than CGS.

Feedback inhibition and recurrent excitation are crucial components of the decision-making network, and disruptions to their operation have been shown to impair the decision-making process [3]. Similarly, synchrony effects are important to a variety of cognitive processes [37] and disease states such as epilepsy [38]. Furthermore, population firing rate distributions follow a characteristic long-tailed structure throughout the brain thought to support sparse neural coding of information [40]. Therefore, although electrical pulses are clearly an effective way to alter decision-making circuits and, more broadly, to interface with the nervous system at large, they may struggle to replicate nuanced interactions that depend on precise spike timing, on-going network activity, and neuron-specific coding. GS generally preserves neural spike-timing, respects on-going network activity, and maintains population firing rate distributions. Recent work has shown that more naturalistic, ‘biomimetic’ intracranial microstimulation (ICMS) outperforms traditional ICMS in somatosensory prosthesis for bionic hands [41]. Since GS mimics the natural cortical firing patterns more closely than PS, we expect it would be a good candidate for improving somatosensory prosthesis, along with any other ICMS application in which ongoing cortical network activity proves to be important.

While there appear to be functional benefits of using GS for cortical micro-stimulation, the safety of using these techniques for clinical use are yet unproven for prolonged duration. The technology that would allow chronic delivery of direct current in an implant is under active investigation [21, 42–46]. At present, the only delivery of direct current that has had clinical validation is tDCS which lacks spatial precision and is physically difficult to develop for practical continual applications [17].

The future of effective cortical neuromodulation technology that would replicate normal function may lie in using the combination of the two methods. While GS can bias the network in a more natural way, PS can be used to deliver more tightly localized and precise neural responses.

## Supplementary Material

Supplementary Material

## Data Availability

The data that support the findings of this study are openly available at the following URL/DOI: https://github.com/pauladkisson/bam_microstim2.
