# Supplementary material for: Galvanic vs. pulsatile effects on decision-making networks: reshaping the neural activation landscape
Source: J Neural Eng. Author manuscript; Available in PMC 2025 Mar 4. (PMC11877455; doi:10.1088/1741-2552/ad36e2)
Supplement: Supplementary Material [file NIHMS2048360-supplement-Supplementary_Material.pdf]

Table S1. LIF Model Parameters

Name / Description	Symbol	Value (pyramidal, interneuron where applicable)
Leak Conductance	g_L	25nS, 20nS
Leak Potential	E_L	-70mV
Membrane Capacitance	C_m	0.5nF, 0.2nF
Threshold Potential	$V_{threshold}$	-50mV
Reset Potential	V_r	-55mV
Refractory Period	τ_r	2ms, 1ms

Table S2. Biophysical Attractor Network Parameters

Name / Description	Symbol	Value (pyramidal, interneuron where applicable)
Number of Neurons	N	1000
Number of P1 Neurons	N_{P1}	120
Number of P2 Neurons	N_{P2}	120
Number of NS Neurons	N_{NS}	560
Number of Int Neurons	N_{Int}	200
Number of Excitatory Neurons	N_E	800
Strong Connection Weight	w_{strong}	1.7
Weak Connection Weight	w_{weak}	0.8765
Medium Connection Weight	w_{medium}	1
Background Input	FR_{bg}	2400Hz
Simulation Time Step	dt	0.05ms

Table S3. Synaptic Parameters

Name / Description	Symbol	Value (pyramidal, interneuron where applicable)
External (Background or Task) AMPA Conductance	$g_{AMPA_{ext}}$	2.1nS, 1.62nS
Recurrent AMPA Conductance	$g_{AMPA_{rec}}$	0.1nS, 0.08nS
AMPA Equilibrium Potential	E_{AMPA}	0mV
AMPA Decay Time Constant	τ_{AMPA}	2ms
GABA Conductance	g_{GABA}	2.6nS, 2nS
GABA Equilibrium Potential	E_{GABA}	-70mV
GABA Decay Time Constant	τ_{GABA}	5ms
NMDA Conductance	g_{NMDA}	0.33nS, 0.26nS
NMDA Equilibrium Potential	E_{NMDA}	0mV
NMDA Rise Time Constant	τ_{NMDA_1}	2ms
NMDA Decay Time Constant	τ_{NMDA_2}	100ms
Extracellular Magnesium Concentration	C_{Mg}	1mM
NMDA Conductance Transition Rate	α	0.5ms^{-1}

Cable Equation Model

Cable equation neurons are modeled by

$$\frac{dV_m^{(k)}}{dt} = \frac{G_a\left(V_m^{(k-1)} - 2V_m^{(k)} + V_m^{(k+1)} + V_{ext}^{(k-1)} - 2V_{ext}^{(k)} + V_{ext}^{(k+1)}\right) - I_{ionic}^{(k)}}{C_m}$$

$$G_a = \frac{\pi d^2}{4\rho_i \Delta x}, \qquad C_m = c_m \pi dL$$

$$I_{ionic}^{(k)} = \pi dL\left(g_{Na}m^3h\left(V_m^{(k)} - E_{Na}\right) + g_K n^4\left(V_m^{(k)} - E_K\right) + g_l\left(V_m^{(k)} - E_l\right)\right)$$

$$\frac{dm^{(k)}}{dt} = \alpha_m\left(V_m^{(k)}\right)\left(1 - m^{(k)}\right) - \beta_m\left(V_m^{(k)}\right)m^{(k)}$$

$$\frac{dh^{(k)}}{dt} = \alpha_h\left(V_m^{(k)}\right)\left(1 - h^{(k)}\right) - \beta_h\left(V_m^{(k)}\right)h^{(k)}$$

$$\frac{dn^{(k)}}{dt} = \alpha_n\left(V_m^{(k)}\right)\left(1 - n^{(k)}\right) - \beta_n\left(V_m^{(k)}\right)n^{(k)}$$

$$\alpha_m(V) = \frac{0.1(V+40)}{1 - e^{-\frac{(V+40)}{10}}}, \qquad \beta_m(V) = 4e^{-\frac{V+65}{18}};\ V \text{ in mV}$$

$$\alpha_h(V) = 0.07e^{-\frac{V+65}{20}}, \qquad \beta_h(V) = \frac{1}{1 + e^{-\frac{V+35}{10}}};\ V \text{ in mV}$$

$$\alpha_n(V) = \frac{0.01(V+55)}{1 - e^{-\frac{(V+55)}{10}}}, \qquad \beta_n(V) = 0.125e^{-\frac{V+65}{80}};\ V \text{ in mV}$$

Where $k = 1, 2, \ldots, N_k$ is the spatial index into the number of spatial nodes N_k. With open boundary conditions:

$$V_m^{(k=0)} = V_m^{(k=1)}, \quad V_{ext}^{(k=0)} = V_{ext}^{(k=1)}, \quad V_m^{(k=N_k+1)} = V_m^{(k=N_k)}, \quad V_{ext}^{(k=N_k+1)} = V_{ext}^{(k=N_k)} \rightarrow$$

$$\frac{dV_m^{(k=1)}}{dt} = \frac{G_a\left(V_m^{(k=2)} - V_m^{(k=1)} + V_{ext}^{(k=2)} - V_{ext}^{(k=1)}\right) - I_{ionic}^{(k=1)}}{C_m}$$

$$\frac{dV_m^{(k=N_k)}}{dt} = \frac{G_a\left(V_m^{(k=N_k-1)} - V_m^{(k=N_k)} + V_{ext}^{(k=N_k-1)} - V_{ext}^{(k=N_k)}\right) - I_{ionic}^{(k=N_k)}}{C_m}$$

Extracellular Voltage Model

The electrical current at the electrode $I_{electrode}$ creates an electric field around the electrode, such that extracellular voltage at any point distance r from the electrode is given by

$$V_{ext}(r) = \frac{\rho_{ext}}{4\pi r} I_{electrode}$$

With respect to a linear axon and point electrode, the distance r can be decomposed into an axial component x and a radial component z such that

$$r = \sqrt{x^2 + z^2}$$

Then, the average extracellular voltage among all the spatial nodes of the axon is

$$\overline{V_{ext}} = \left\langle \frac{\rho_{ext}}{4\pi\sqrt{x^2 + z^2}} I_{electrode} \right\rangle_x$$

$$x = \Delta x n$$

$$n = -\left(\frac{N_k - 1}{2}\right), -\left(\frac{N_k - 3}{2}\right), \dots, 0, 1, \dots, \left(\frac{N_k - 1}{2}\right)$$

Where N_k is the number of spatial nodes, and Δx is the internode distance.

Table S4. Cable Equation Model Parameters

**Name / Description**	**Symbol**	**Value (pyramidal, interneuron where applicable)**
Axon Diameter	d	7μm
Node Length	L	2.5μm
Internode Distance	Δx	0.1cm
Intracellular Resistivity	ρ_i	0.055kΩ-cm
Specific Membrane Capacitance	c_m	1μF/cm^2
Sodium Specific Conductance	g_{Na}	120mS/cm^2
Sodium Equilibrium Potential	E_{Na}	66.60076227mV
Potassium Specific Conductance	g_K	36mS/cm^2
Potassium Equilibrium Potential	E_K	-89.0621027mV
Leak Channel Specific Conductance	g_l	0.3mS/cm^2
Leak Channel Equilibrium Potential	E_l	-70.51124916mV
Number of Spatial Nodes	N_k	25
Extracellular Resistivity	ρ_{ext}	0.3kΩ-cm

Table S5. Pulsatile and Galvanic Stimulation Parameters

Name / Description	Symbol	Value (pyramidal, interneuron where applicable)
Pulse Amplitude	$I_{electrode}$	10μA
Pulse Rate	r_{pulse}	200Hz
Pulse Width	w_{pulse}	300μs/phase
LIF Pulse Constant	k_{ps}	5.25nS, 4.2nS
Galvanic Amplitude	$I_{electrode}$	1.4μA
Maximum Injected Current	I_{max}	1135pA
LIF Galvanic Constant	k_{gs}	25nS, 20nS

Table S6. Pulsatile Refractory Parameters

Electrode Current Amplitude (μA)	Pulse-Pulse Refractory Period (t_{pp}, ms)	Pulse-Spontaneous Refractory Period (t_{ps}, ms)
0	1	0
4.5	111	1
9.0	155	1
13.5	119	1
18.0	52	1
22.5	18	7
27.0	14	10
31.5	13	10
36.0	11	10
40.5	8	10
45.0	7	10
49.5	6	10
54.0	5	10
58.5	5	10
63.0	5	10
67.5	5	10
72.0	5	10
76.5	5	10
81.0	4	10
85.5	4	10
90.0	4	10
96.0	4	19
108.0	4	19

120.0	4	19
132.0	5	19
144.0	5	19
156.0	5	19
168.0	5	19
180.0	5	19
192.0	5	19
204.0	5	19
216.0	5	19
228.0	5	19
240.0	5	19
252.0	5	19
264.0	6	19
276.0	6	19
288.0	7	19
300.0	7	19
312.0	9	29
324.0	10	54
336.0	83	80
348.0	116	100
360.0	116	100
500.0	132	100

Table S7. Task Related Input Coherence

Pulsatile Stimulation (%)	Cathodic Galvanic Stimulation (%)	Anodic Galvanic Stimulation (%)	No Stimulation (%)
-100	-100	-100	-100
-82.6	-82.6	-70	-51.2
-69.8	-69.8	-21.2	-25.6
-63.4	-63.4	0	-12.8
-57.0	-57.0	4.4	-6.4
-53.8	-53.8	17.2	-3.2
-50.6	-50.6	23.6	0
-44.2	-44.2	26.8	3.2
-31.4	-31.4	30	6.4
-5.8	-5.8	33.2	12.8
0	0	36.4	25.6
43	43	42.8	51.2
100	100	55.6	100
-	-	81.2	-
-	-	100	-
